# Water‐related innovations in land plants evolved by different patterns of gene cooption and novelty

**DOI:** 10.1111/nph.17981

**Published:** 2022-02-08

**Authors:** Alexander M. C. Bowles, Jordi Paps, Ulrike Bechtold

**Affiliations:** ^1^ School of Life Sciences University of Essex Wivenhoe Park Colchester CO4 3SQ UK; ^2^ School of Geographical Sciences University of Bristol University Road Bristol BS8 1RL UK; ^3^ School of Biological Sciences University of Bristol 24 Tyndall Avenue Bristol BS8 1TQ UK; ^4^ Present address: Department of Biosciences Durham University South Road Durham DH1 3LE UK

**Keywords:** comparative genomics, plant evolution, roots, stomata, vascular tissue

## Abstract

- The origin of land plants and their descendants was marked by the evolution of key adaptations to life in terrestrial environments such as roots, vascular tissue and stomata. Though these innovations are well characterized, the evolution of the genetic toolkit underlying their development and function is poorly understood.
- We analysed molecular data from 532 species to investigate the evolutionary origin and diversification of genes involved in the development and regulation of these adaptations.
- We show that novel genes in the first land plants led to the single origin of stomata, but the stomatal closure of seed plants resulted from later gene expansions. By contrast, the major mechanism leading to the origin of vascular tissue was cooption of genes that emerged in the first land plants, enabling continuous water transport throughout the ancestral vascular plant. In turn, new key genes in the ancestors of plants with true leaves and seed plants led to the emergence of roots and lateral roots.
- The analysis highlights the different modes of evolution that enabled plants to conquer land, suggesting that gene expansion and cooption are the most common mechanisms of biological innovation in plant evolutionary history.

## Abstract 2

See also the Commentary on this article by Fürst‐Jansen *et al.*, **235**: 380–383.

## Introduction

**Fig. 1:**
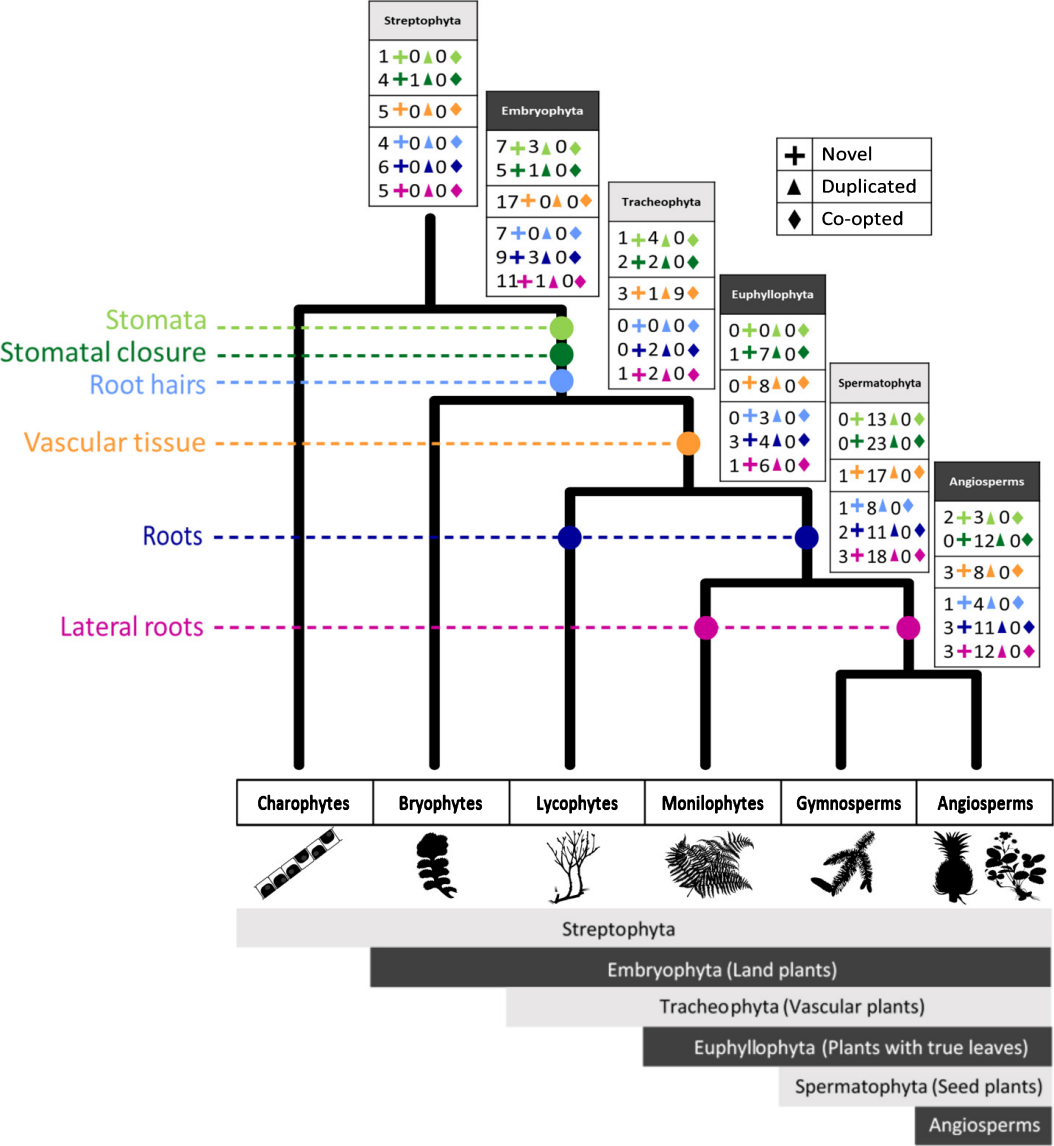
Plant–water relationships have evolved in a stepwise manner. The tree demonstrates the evolutionary relationships of plants with silhouettes below (sourced from phylopic.org) illustrating species in each group. The dashed lines leading to each different water regulatory innovation denote their origins. The colour is repeated within the boxes illustrating the genetic mechanisms associated with each innovation (light blue: stomatal development, dark blue: stomatal signalling, orange: vascular tissue development, light green: root hair, green: primary root, dark green: lateral root development). The key demonstrates the numbers of novel, expanded and coopted genes in the boxes. The major plant groups are classified at the bottom in the solid block colour (e.g. Embryophyta, Tracheophyta) and their common names are listed in the dashed boxes beside (e.g. Land plants, Vascular plants).

The first land plants and their descendants have adapted to a multitude of new environments (Morris *et al*., 2018). Genome analysis has identified that gene homologues for major biological innovations often precede terrestrialization, suggesting that innovations once thought to be land plant‐specific may have emerged in older ancestors (e.g. associations with substrate microbiota) (Hori *et al*., 2014; Nishiyama *et al*., 2018; Wang *et al*., 2019; Bowles *et al*., 2020). Though many genes evolved before the plant transition to land, genetic rewiring of developmental and stress response pathways occurred later in plant evolution, increasing the adaptive plasticity to water availability (Fürst‐Jansen *et al*., 2020). Innovations important for water regulation and transport have evolved in a stepwise manner (Fig. 1). These include the evolution of the cuticle in the ancestor of land plants which acts as an extracellular hydrophobic barrier providing desiccation protection, whilst convergent evolution of leaves within vascular plants (in lycophytes, ferns and seed plants) refined the control of water movement (Harrison, 2017). Three of the most important features for water regulation, and the focus of this study, are stomata, vascular tissue and roots.

Stomata, a key adaptation to dry environments, are pores in plant tissue that enable gas exchange and that regulate water loss (Sussmilch *et al*., 2019). The origin of stomata is a key adaptation of the first land plants, enabling plants to proliferate in terrestrial environments (Chater *et al*., 2017). The aperture of stomata is regulated by multiple factors including light, atmospheric CO_2_ and abscisic acid (ABA) levels (Araújo *et al*., 2011). Under drought stress, ABA accumulates, triggering a signalling pathway that leads to stomatal closure (Bharath *et al*., 2021). This control enabled ancestral plants to optimize the balance between CO_2_ uptake and water loss.

Although stomata emerged in the ancestor of land plants, stomatal function shows clear distinctions between different plant lineages. There is debate about the emergence of active stomatal closure, in which hormonal or environmental cues trigger a network of signalling proteins regulating stomatal aperture (Plackett *et al*., 2021). Comparative analyses have suggested that ancestral land plants possessed actively controlled stomata (Harris *et al*., 2020). Early evidence from physiological studies contradicts this, suggesting that stomata closure of vascular plants was hydropassive (Brodribb & McAdam, 2011). However, further physiological studies suggest that lycophytes and ferns do respond to experimentally introduced ABA (Ruszala *et al*., 2011; Chater *et al*., 2016; Cai *et al*., 2017), although studies often used nonphysiological levels of ABA (Brodribb & McAdam, 2017). Regardless of where active stomatal closure emerges, it is undisputed that there are evolutionary distinctions between the stomatal control of distinct plant groups, with seed plant stomata responding rapidly to water availability (Brodribb & McAdam, 2017). This draws into question the origins of stomatal closure in the evolutionary history of plants.

In tracheophytes, also known as vascular plants, xylem and phloem tissues transport water through the plant, which is then evaporated through stomata (Lucas *et al*., 2013). Lignified vascular tissue also provides mechanical support enabling plants to increase their size and stature and to dominate terrestrial habitats. Indeed, the first trees in the fossil record are early vascular plants (Stein *et al*., 2007). The current understanding of vascular tissue development reveals a complex and highly coordinated set of genes controlling the specification, patterning and growth of distinct cell types (De Rybel *et al*., 2016). However, due to this complexity, the evolutionary origin of these genes is poorly understood.

Rooting systems provide multiple functions including anchorage and water uptake (Raven & Edwards, 2001). Distinct components of the rooting system emerged at different points during plant evolution; root hairs were present in the first vascular plants (Kenrick & Strullu‐Derrien, 2014), primary roots emerged convergently in lycophytes and plants with true leaves (Hetherington & Dolan, 2018), whilst lateral roots emerged convergently in ferns and seed plants (Hetherington *et al*., 2020). The first land plants had a rhizoid‐based rooting system that has been distinguished from the root hairs of vascular plants (Kenrick & Strullu‐Derrien, 2014). Sequence and functional homology have been identified for root hair development genes in the rhizoid development of *Physcomitrium patens*, but the structural homology between rhizoids and root hairs is unclear (Menand *et al*., 2007; Tam *et al*., 2015; Honkanen *et al*., 2016). The piecemeal and convergent evolution of the rooting system is underpinned by a toolkit of genes whose origin and molecular evolution is poorly understood.

As mentioned above, the evolution of these three innovations, namely roots, vascular tissue and stomata, is underpinned by plant genome evolution. As recently highlighted by the one thousand plant transcriptomes (1KP) project, which analysed patterns of gene diversification for the 23 largest plant gene families, ancestral streptophyte and embryophyte genomes were characterized by gene novelty whilst the genomes of younger plant ancestors were distinguished by gene and genome duplication (Leebens‐Mack *et al*., 2019). These patterns of novelty are also observed genome‐wide by comparative genomic analyses using complete genomes (Bowles *et al*., 2020). However, not much is known about the mode of evolution – gene novelty vs expansion vs cooption – of the genetic components involved in stomata, vascular tissue and roots. Understanding the evolution of key developmental and signalling genes is the first step in reconstructing the evolutionary history of these innovations. Here, to investigate the modes of genome evolution of important features for adapting to life on land, we use a comparative approach, incorporating genomic and transcriptomic data representing 532 species from across the plant tree of life. Our results show that different patterns of genome evolution drive the emergence of each of the genetic toolkits of stomata, vascular tissue and roots.

## Materials and Methods

### Homology assignment

The pipeline approach has previously been described and used to investigate genome evolution (Paps & Holland, 2018; Bowles *et al*., 2020; Guijarro‐Clarke *et al*., 2020). Briefly, proteins were extracted for 208 genomes, including 178 land plant species and broad outgroup sampling, and similarity between proteins was identified with an all‐vs‐all Blast search (Altschul *et al*., 1990). Sequences were clustered into Homology Groups (HGs) using Markov Clustering (MCL) with a granularity score of 2 (Enright *et al*., 2002). MCL uses graph theory and hidden Markov models to cluster proteins into groups based on the output of the Blast comparisons. These HGs will contain large gene families, with orthologues and paralogues. This method does have limitations shared with similar Blast‐based approaches, including the lack of detection of lateral gene transfer or gene fusion/fissions. Further division of the HGs (e.g. split into orthologues and paralogues) was not pursued at this stage, instead being completed by gene tree analysis as described below.

### Expanding taxon sampling

To accommodate the ever‐growing representation of plant omics data, the taxon sampling was expanded beyond our original sampling of 208 complete genomes (Bowles *et al*., 2020). First, data from the 1KP project were sourced and binned into seven local Blast databases (charophytes, hornworts, liverworts, mosses, lycophytes, ferns, gymnosperms) (Leebens‐Mack *et al*., 2019). Second, Blast databases for newly published evolutionary significant genomes were constructed, grouped into streptophytes (Supporting Information Table S1; Dataset S1): *Chlorokybus atmophyticus*, *Mesostigma viride* (Wang *et al*., 2019), *Chara braunii* (Nishiyama *et al*., 2018), *Penium margaritaceum* (Jiao *et al*., 2020), *Spirogloea muscicola* and *Mesotaenium endlicherianum* (Cheng *et al*., 2019); bryophytes: *Anthoceros angustus* (Zhang *et al*., 2020), *Anthoceros agrestis*, *Anthoceros punctactus* (Li *et al*., 2020), *Fontinalis antipyretica* (Yu *et al*., 2020a) and *Pleurozium schreberi* (Pederson *et al*., 2019); ferns: *Azolla filiculoides* and *Salvinia cucullata* (Li *et al*., 2018); and gymnosperms: *Abies alba* (Mosca *et al*., 2019), *Picea glauca* (Warren *et al*., 2015), *Picea lambertiana* (Stevens *et al*., 2016), *Picea taeda* (Zimin *et al*., 2014), *Pseudotsuga menziesii* (Neale *et al*., 2017) and *Sequoiadendron giganteum* (Scott *et al*., 2020). The *Arabidopsis thaliana* genes from each HG inferred from the 208 genomes dataset were extracted and searched against each of these databases using reciprocal best‐hit Blast, specifying a maximum of 20 species per Blast database (Tatusov *et al*., 1997). Therefore, in this analysis, in total, 227 genomes and 305 nonangiosperm plant transcriptomes were analysed, representing all major streptophyte lineages.

### Genetic toolkit of stomatal development and signalling, vascular tissue development and root development

The literature was searched to identify genes involved in developmental and signalling pathways of stomata, vascular tissue and roots. For stomatal development genes, a composite list was made from Lau & Bergmann (2012); Le *et al*. (2014) and Chater *et al*. (2017). Stomatal signalling genes were identified from Cotelle & Leonhardt (2016); Albert *et al*. (2017) and Cai *et al*. (2017). For the development of vascular tissues, genes from Ruonala *et al*. (2017) were used. Root development genes were identified in Jung & McCouch (2013) whilst root hair development genes were identified in Vissenberg *et al*. (2020). For lateral root development, genes from Verstraeten *et al*. (2014); Oh *et al*. (2018) and Santos Teixeira *et al*. (2019) were used.

In the previous research, the 208 genomes dataset was queried based solely on taxonomic occupancy (Bowles *et al*., 2020). New to this study, the dataset was queried for the list of candidate genes compiled above. Specifically, HGs were extracted based on the Uniprot IDs for each gene using a new computational script, MCL_search_by_gene_name_2.pl (Dataset S2) (UniProt Consortium, 2018). Note that gene counts for individual species may be overinflated by the merging of sequences with subspecies data and by multiple systems of nomenclature used during gene annotation. Information about the accession and sequence details for all HGs is available in Dataset S3, as discussed below.

### Detecting mechanisms of gene evolution

Three evolutionarily distinct mechanisms of gene evolution were subsequently identified. Novel HGs are here defined as a set of genes present in the last common ancestor (LCA) of a clade and absent in all outgroups; in the context of the investigated innovations, the novel genes and their related innovations emerge in the same node of the evolutionary tree. A coopted HG is defined as a set of genes traditionally associated with a biological innovation (e.g. vascular tissue) whose origin pre‐dates the emergence of such innovation (e.g. HG originating in the LCA of land plants linked to vascular tissue development). To investigate the diversification of genes, gene phylogenies were inferred and examined (see next section; Dataset S3). From this, an expanded HG was defined as a group of genes that underwent gene duplications in the LCA of the clade of interest linked to a biological innovation.

### Gene tree inference

A curated list of species in the original genomic dataset was collated with representatives for each major plant group. These were *Cyanidioschyzon merolae* (Rhodophyta), *Cyanophora paradoxa* (Glaucophyta), *Bathycoccus prasinos* (Chlorophyta), *Klebsormidium flaccidum* (now called *K. nitens*, Charophyta), *Marchantia polymorpha*, *Physcomitrium patens* (bryophytes), *Selaginella moellendorffii* (Lycophyta), *Picea abies* (gymnosperms), *Amborella trichopoda* (ANA grade), *Oryza sativa indica*, *Brachypodium distachyon* (monocots) and *A. thaliana* (eudicots). These HGs were extracted using the MCL_search_by_gene_name_2.pl script (Dataset S2) (UniProt Consortium, 2018). A file containing all gene IDs for all species was then used to extract the protein sequences using the perl one liner ‘perl ‐ne 'if(/^>(\S+)/){$c=$i{$1}}$c?print:chomp;$i{$_}=1 if @ARGV' ids.file all_fasta.file’. To each HG fasta file, the sequences from the reciprocal Blast queries were added, incorporating data from the 1KP transcriptomes and additionally published genomes (Dataset S1).

To investigate expanded HGs, sequences from each HG were aligned using Mafft using the –auto parameter, which automatically selects an appropriate alignment strategy as well as the leave gappy region parameter (Katoh *et al*., 2002). Multiple sequence alignments were trimmed with Trimal using the gappyout method, to identify and remove poorly aligned positions. Gene trees (bootstrapped maximum‐likelihood phylogenies) were inferred using Iq‐Tree, using the inbuilt ModelFinder (MFP) to select the best‐fitting substitution model, specifying 1000 ultrafast bootstrap replicates (Nguyen *et al*., 2015). Trees were rooted against the latest plant phylogeny to infer gene innovations, expansions and cooptions. Trees were visualized in iTOL (Letunic & Bork, 2019) and are available at https://itol.embl.de/shared/Bowles_et_al.

## Results

### Gene novelty suggests that stomata evolved once in the ancestor of land plants

**Fig. 2:**
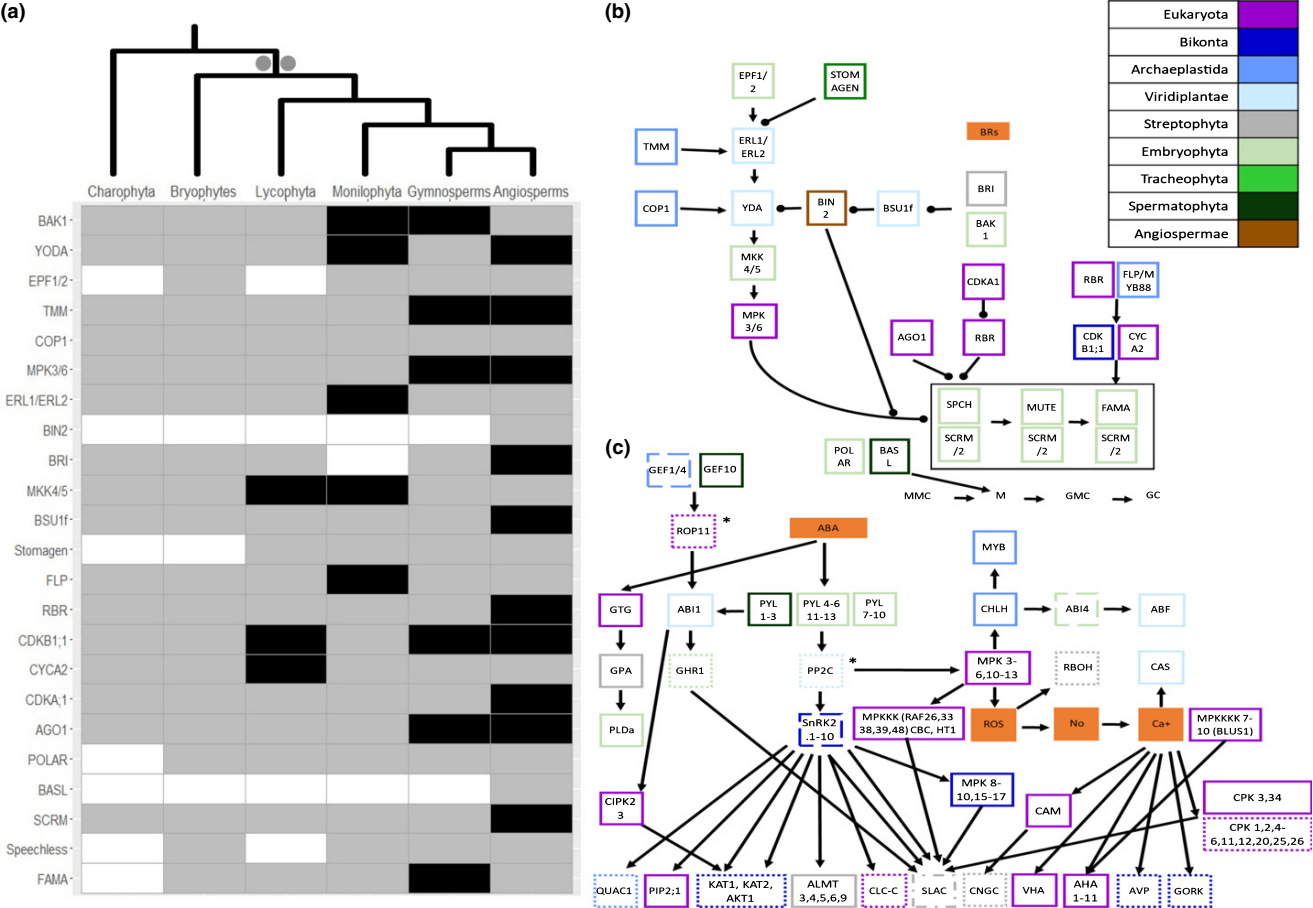
The genomic basis of the evolutionary development of stomata and stomata signalling. (a) Heatmap displaying absence (white), partial presence (grey) and presence (black) in all species for both transcriptomic and genomic data for genes involved in stomatal development (Supporting Information Figs S1, S2). The tree at the top illustrates plant evolutionary relationships and the two grey dots denote the origin of stomata and stomata signalling (grey). (b) Genetic network leading to the development of stomata. Each gene is coloured based on its phylogenetic appearance. Nongenetic components are coloured in solid orange. Lines ending in circles denote negative interactions whilst lines ending in arrows denote activation. The states of guard cell development are highlighted: MMC (meristemoid mother cell), M (meristemoid), GMC (guard mother cell) and GC (guard cell). (c) Genetic network involved in stomatal signalling. Expanded genes in the ancestor of Spermatophyta are highlighted by boxes with dotted edges. Expanded genes in the ancestor of Euphyllophyta are highlighted by boxes with dashed edges. Expanded genes in the ancestor of Tracheophyta are highlighted by boxes with dot–dash–dot edges. Asterisks indicate an HG that expands twice, once in the ancestor of Euphyllophyta and again in the ancestor of Spermatophyta. Nongenetic components are coloured in solid orange. Lines ending in circles denote negative interactions whilst lines ending in arrows denote activation.

Stomata in bryophytes (liverworts, mosses and hornworts) demonstrate a patchy distribution with absences in liverworts and some mosses, but are found in all vascular plants (Duckett & Pressel, 2018). This raises questions about the single origin of stomata in the first land plants or their convergent evolution in the ancestors of vascular plants, mosses and hornworts. Here, our analysis shows the stomatal development pathway originated in the LCA of land plants, based on inferences from a taxonomically broad representation of genomes and transcriptomes. Of the 23 stomatal development genes, 21 pre‐dated or accompanied the origin of land plants (Figs 2a,b, S1; Dataset S2). In *A. thaliana*, the basic helix–loop–helix (bHLH) genes SPCH, MUTE and FAMA are required consecutively to determine stomatal development, aided by the bHLH transcription factor SCREAM (Fig. 2a,b). In our analysis, SCREAM was identified in all land plants, even the liverwort *M. polymorpha*, which does not have stomata. MUTE, SPCH and FAMA were present in the LCA of land plants but were not recovered in *M*. *polymorpha*, potentially accompanying stomatal loss in liverworts, as previously observed (Chater *et al*., 2017). The evolutionary age of these 21 stomatal genes suggests that bryophyte stomata may develop in a similar manner to vascular plant stomata, and by extension the stomata of the first land plants. STOMAGEN, which regulates stomatal density (Sugano *et al*., 2010), appears in the origin of vascular plants, the first time stomata appear on leaf‐like structures (Fig. 2a,b). Of the three features investigated, stomata were the only innovation with a strong association with novel genes.

### Gene expansion enabled stomatal control in the ancestor of seed plants

Gene families involved in stomatal closure experienced multiple gene duplications, predominantly in the ancestor of seed plants, which could precede the neo‐ or subfunctionalization of the duplicated genes (Figs 2c, S2; Dataset S3). Genes involved in the signalling of some potassium and anion channels (QUAC1, KAT2, AKT1, GORK) were present before the origin of land plants and expanded in the ancestor of seed plants (Fig. 2; Dataset S3). Additionally, important for stomatal closure is a group of PYLs (1–3) (PYR1‐LIKE 1), an ABA receptor, which emerged in the ancestor of seed plants (Fig. 2). The remaining PYLs (4–13) emerged in the ancestor of land plants (Fig. 2c).

Although most gene expansions were identified in the LCA of seed plants, several notable expansions were found in the ancestor of plants with true leaves. One such example is SNRK2 (SNF1‐RELATED PROTEIN KINASE 2), a family of genes including OST1 (SNRK2.6), which is a core element of the ABA=dependent signalling pathway (Fig. 2c). Furthermore, an HG containing PP2Cs (Protein Phosphatase 2Cs), which regulate ABA‐dependent activation, expanded in the ancestor of plants with true leaves (Fig. 2c). Our data specifically highlight the genetic rewiring of the PP2C–SnRK2 complex as the central regulator of ABA signalling in the ancestor of plants with true leaves. These findings suggest that gene expansions, especially in ABA signalling pathways, played an essential role in the evolution of stomatal closure, allowing plants to preserve water by restricting transpiration rates. These may be the product of whole genome duplications in the ancestor of seed plants (Leebens‐Mack *et al*., 2019) or frequent gene duplication (Ezoe *et al*., 2020).

### Vascular tissue evolved through a complex of genetic mechanisms

**Fig. 3:**
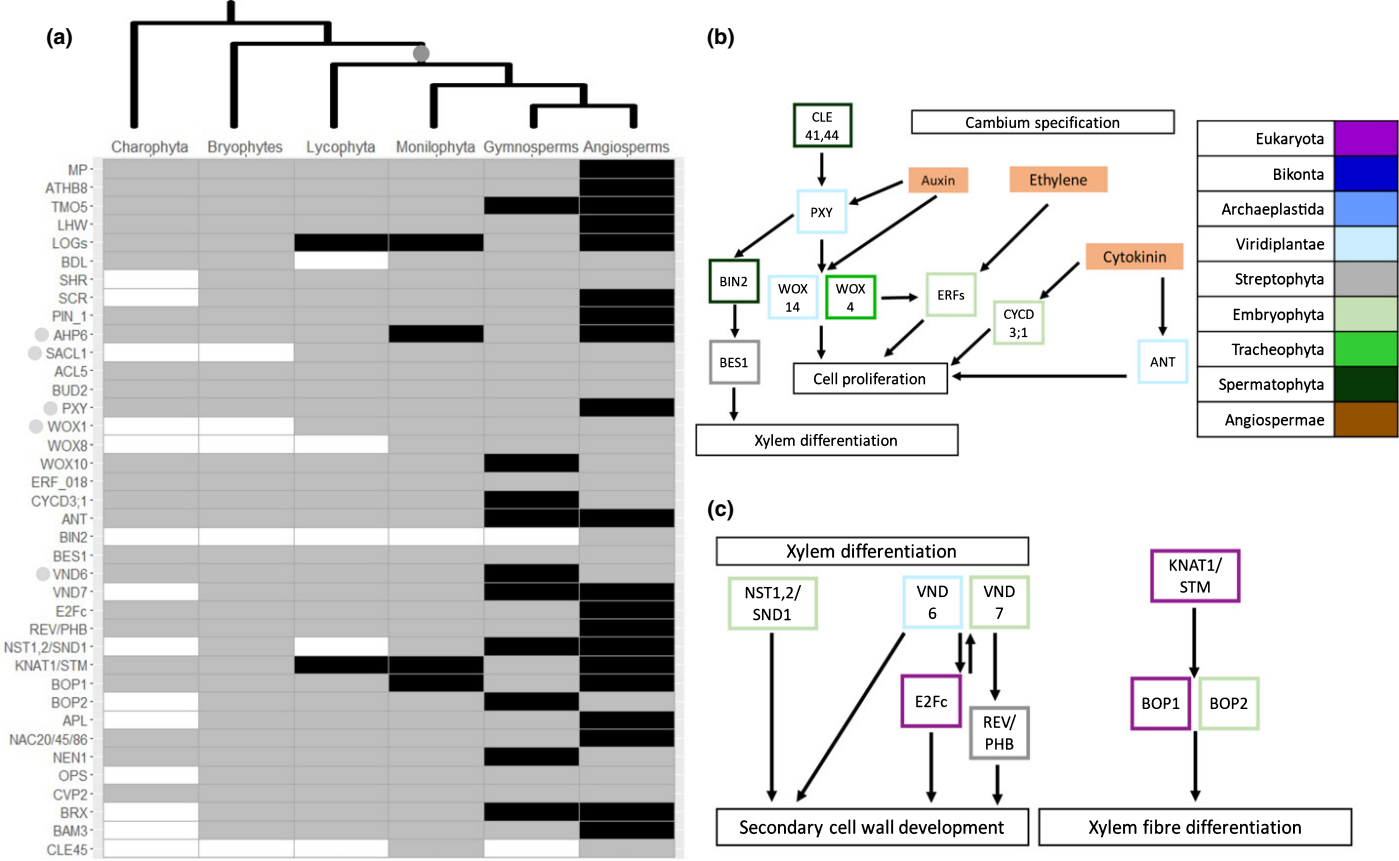
The genomic basis of the evolutionary development of vascular tissue. (a) Heatmap displaying absence (white), partial presence (grey) and presence (black) in all species for both transcriptomic and genomic data for genes involved in vascular tissue development (Supporting Information Fig. S3). The tree at the top illustrates plant evolutionary relationships and the origin of vascular tissue (grey). Genes discussed in the text are highlighted by a grey dot. (b) Genetic network involved in cambium specification. Each gene, in panels (b) and (c), is coloured based on its phylogenetic appearance. Expanded genes are highlighted by boxes with dotted edges and coopted genes are highlighted by boxes with dashed edges. Lines ending in circles denote negative interactions whilst lines ending in arrows denote activation. (c) Genetic network involved in xylem differentiation. Nongenetic components are coloured in solid orange. Lines ending in circles denote negative interactions whilst lines ending in arrows denote activation.

Only two HGs (SACL1: SUPPRESSOR OF ACAULIS 51; and WOX1: WUSCHEL‐related homeobox 1) involved in vascular tissue development originated in the ancestor of vascular plants (Figs 3a, S3; Dataset S3). Several genes involved in vascular tissue development emerged in the ancestor of land plants, showing a patchy distribution in nonvascular plants but being present in all the vascular plant species sampled in our analyses (176 tracheophyte genomes). This retention in the LCA of vascular plants suggests a vital function in the biology of this group. Nine HGs fitted this criterion of cooption (Fig. 1). These HGs were found across all vascular development pathways, suggesting that vascular tissue emerged through a complex of evolutionary mechanisms. These findings demonstrate the importance of gene expansions and repurposing of old genes for novel functions in the evolutionary development of the vascular system. Together these genetic processes contributed to the origin and evolution of one of the most successful plant groups, *c*. 450 million yr ago (Morris *et al*., 2018).

### Novel genes enabled the evolution of roots

**Fig. 4:**
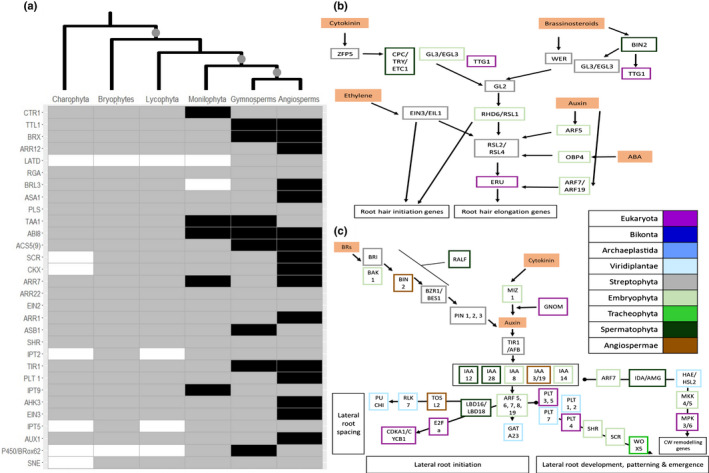
The genomic basis of the evolutionary development of roots, root hairs and lateral roots. (a) Heatmap displaying absence (white), partial presence (grey) and presence (black) in all species for both transcriptomic and genomic data for genes involved in primary root development (Supporting Information Figs S4–S6). The tree at the top illustrates plant evolutionary relationships and the grey dots denote the origin of root hairs, roots and lateral roots. (b) Genetic network leading to the development of root hairs. Each gene, in Fig. 2(b,c), is coloured based on its phylogenetic appearance. Nongenetic components are coloured in solid orange. Lines ending in circles denote negative interactions whilst lines ending in arrows denote activation. (c) Genetic network leading to the development of lateral roots. Lines ending in circles denote negative interactions whilst lines ending in arrows denote activation.

Our analyses show that novel genes in land plants enabled the evolutionary development of root hairs (Figs 4, S4). CAPRICE (CPC), TRIPTYCHON (TRY) and ENHANCER OF TRY AND CPC 1 (ETC1) are together in a single HG and emerge in the ancestor of seed plants, to promote root hair cell differentiation. This suggests that nonseed plants develop root hairs without these genes and that greater control of development evolved in the LCA of seed plants.

Fossil evidence supports at least two origins of roots in the evolutionary history of plants, once in the ancestor of lycophytes and again in the ancestor of plants with true leaves (Hetherington & Dolan, 2018). Analysis of genes involved in primary root development revealed that many were conserved across land plants (Figs 4a, S5). Two HGs, ARABIDOPSIS RESPONSE REGULATOR 12 (ARR12) and LATERAL ROOT ORGAN DEFECTIVE (LATD), have emerged in the LCA of euphyllophytes (Dataset S4). Both HGs have been shown to modulate primary and lateral root growth and development, with responses to ABA and water deprivation (Léran *et al*., 2014; Nguyen *et al*., 2016).

The data demonstrate that the majority of lateral root development genes pre‐date the emergence of lateral roots, originating in the ancestors of Streptophyta (e.g. PIN1–3) and land plants (e.g. IAA 8, 14), which contribute to other functions in these rootless plants (Mutte *et al*., 2018). Three key genes appeared with the origin of lateral roots in the ancestor of seed plants: INDOLEACETIC ACID‐INDUCED PROTEIN 12 and 28 (IAA12, 28), INFLORESCENCE DEFICIENT IN ABSCISSION (IDA) and RAPID ALKALINIZATION FACTOR (RALF; Dataset S4). These three HGs are involved in multiple stages of lateral root development, suggesting their emergence contributed to the origin of lateral roots.

Hydrotropism is the directional growth of plants towards water, enabling acquisition of water under drought stress (Dietrich *et al*., 2017), an important target trait for drought avoidance. The two key genes essential for hydrotropism are MIZU‐KUSSEI 1 (MIZ1) and MIZU‐KUSSEI 2 (MIZ2/GNOM). The data presented infer that MIZ1 emerged and diversified through expansion in the ancestor of land plants (Fig. 4c; Dataset S3). This suggests that rooting system hydrotropism may have been important during plant terrestrialization (Shkolnik *et al*., 2018). The HG containing MIZ2/GNOM was present in the ancestor of eukaryotes, with genes expanding in the ancestor of seed plants, potentially responsible for lateral root hydrotropism (Fig. 4c; Dataset S3). The development of increasingly morphologically complex rooting and response systems in the ancestors of land plants, plants with true leaves and seed plants has enabled access to previously unavailable water sources. The data presented above demonstrate that a combination of novel genes is responsible for the emergence of plants with true roots with lateral branches that are capable of responding to water gradients in the soil.

## Discussion

Overall, these analyses provide novel insights into the molecular evolution of three morphological innovations fundamental for plant life. By combining these inferences about patterns of gene evolution for stomata, vascular tissue and roots, insights into plant genome evolution more broadly can be garnered. The most striking revelation is that each of these morphological innovations is underpinned by different patterns of gene evolution. We demonstrated that novel genes led to the origin of stomata (Fig. 2a,b) in the first land plants, but stomatal control developed over the course of plant evolutionary history, with gene expansions leading to rapid closure in the first seed plants (Fig. 2c). The development of vascular tissue, conversely, is mostly associated with cooption, the repurposing of old genes for new functions (Fig. 3). Another striking pattern is that, aside from stomata, very few novel genes emerged simultaneously with the morphological innovation with which they were associated (Fig. 1). This suggests that gene expansion and gene cooption are the more common mechanisms of biological innovation throughout plant evolutionary history.

Comparative analysis of the stomatal development pathway recovered most genes as originating in the ancestor of land plants, suggesting that the stomata of the first land plants and extant ones developed in a similar manner. This reinforces studies using transcriptome and other sequence data (Chater *et al*., 2017; Harris *et al*., 2020). Due to the single origin of stomata in the ancestor of land plants, this would suggest that reductive processes contributed to stomatal evolution in bryophytes as identified by Harris *et al*. (2020). As highlighted above, the evolution of stomatal closure has been widely debated. Perhaps most importantly, we find the vast majority of stomatal signalling genes in the LCA of land plants and older ancestors, suggesting an ancestral, conserved function in the land plant ancestor. These inferences are supported by physiological studies of early diverging land plants (Chater *et al*., 2011; Ruszala *et al*., 2011; Cai *et al*., 2017). These findings echo those of Harris *et al*. (2020) who conducted similar work for 18 stomatal function genes using a reduced sequence dataset compared to the 34 genes investigated in this study. A gene shared between these analyses is OST1 (SnRK2.6), which in Harris *et al*. (2020) is found in single copy emerging in the ancestor of Streptophyta. This finding is developed further in this study, which identifies all SnRK2s in a multigene family emerging in the ancestor of Viridiplantae. This is due to the homology approach compared to the orthology approach of Harris *et al*. (2020). Our results suggest that the evolutionary trajectory of stomata has been shaped by gene expansions leading to gene neo‐ or subfunctionalization in younger ancestors. Additional genes investigated include multiple PYLs and SnRK2s, key components in ABA signalling, the latter of which were found to duplicate in the ancestor of plants with true leaves (Fig. 2). This could explain why there is an evolutionary distinction between the stomatal control of plants with true leaves and older ancestors. The data here also support widescale gene expansion in the ancestor of seed plants, suggesting a difference between the stomatal control of seed plants and other plants.

Gene cooption was identified as the major mechanism leading to the evolution of vascular tissue (Fig. 3). This suggests that this new morphological structure was the product of the repurposing of a preexisting genetic toolkit already found in land plants. Importantly, there is recent experimental evidence for the redeployment of several individual genes essential for vascular cell development (e.g. TMO5/LHW; Lu *et al*., 2020), supporting our findings of gene cooption as a major mechanism in vascular tissue development. An alternative hypothesis to the origin of vascular tissue in plants suggests that vascular‐like tissue is present in bryophytes and therefore potentially in the LCA of land plants (Brodribb *et al*., 2020). This is based on the patchy distribution of water‐ and food‐conducting cells found in the bryophytes (Ohtani *et al*., 2017), potentially having a similar evolutionary history to stomata, which have undergone reductive evolution in the bryophytes (Harris *et al*., 2020). Therefore, the coopted genes identified in this study could represent novel land plant genes if vascular‐like tissue was present in the LCA of land plants.

All, bar one, root hair development genes were present in the ancestor of land plants. CPC, TRY and ETC1 are in a single HG and emerge in the ancestor of seed plants, to promote root hair cell differentiation by repressing GLABRA 2 and 3 (Tominaga *et al*., 2008). This suggests that nonspermatophyte land plants develop root hairs without these genes and that greater control of root hair development evolved in the LCA of seed plants. Only two genes, ARR12 and LATD, originated in the ancestor of plants with true leaves, accompanying the origin of primary roots. ARR12 regulates cell differentiation and meristem growth (Yokoyama *et al*., 2007; Moubayidin *et al*., 2010). Under drought, ARR12 is downregulated as an adaptive mechanism to control root growth to cope with water deficit (Nguyen *et al*., 2016). LATD is also required for lateral root and nodule meristem development (Léran *et al*., 2014) and, in concert with ABA, modulates primary root elongation (Zhang *et al*., 2014), thus suggesting a potential mechanism for controlling root growth under water stress conditions. These genes therefore play crucial roles in plant growth and development but also in response to water availability. Three HGs emerged at the same time as lateral roots, IAA12, IDA and RALF. IAA12 and IAA28 are auxin‐responsive proteins important for the production of lateral root primordia and optimizing the distribution of new root organs (De Rybel *et al*., 2010; Stoeckle *et al*., 2018). IDA is required for cell wall dissolution, by facilitating the separation of epidermal tissues, enabling lateral root emergence (Zhu *et al*., 2019). RALF1 inhibits cell elongation for lateral root formation and density, particularly under drought stress (Li *et al*., 2019). Under drought stress, RALF1 modulates root hair growth and cell size, and together with other RALFs is implicated in stress responses of lateral roots (Murphy & De Smet, 2014; Zhu *et al*., 2020). These three HGs are involved sequentially in the initiation and formation of lateral roots, and therefore the emergence of these genes enabled the evolution of lateral roots (Fig. 4a,c).

In this study, we investigated the evolutionary development of stomata, vascular tissue and roots using genes characterized in flowering plants, specifically based on *A. thaliana*. As these genes are involved in a known function in *A. thaliana*, genes in other organisms within an HG were assumed to be functionally homologous. Indeed, several genes have been experimentally demonstrated to have conserved functions across the major plant lineages (i.e. ABA signalling genes in ferns (Cai *et al*., 2017), stomatal development genes in mosses (Caine *et al*., 2020), primary root development in ferns (Yu *et al*., 2020b)). However, there are limitations with using sequence homology as a predictor of gene function (Gabaldón & Koonin, 2013). Whilst comparative genomics can be used to predict functionally analogous genes, experimental validation is needed to fully characterize gene function. In spite of these caveats, this study utilizes genome‐scale data from the largest plant genome study to date (Bowles *et al*., 2020), incorporating additional transcriptomic and genomic data providing broad taxonomic coverage for all major groups, to investigate the molecular evolution of the relationship of plants with water.

The ancestor of land plants had a very limited ability to regulate water content. The evolution of stomata, vascular tissue and roots increased the capacity of water transport and regulation of seed plants. The development of these features at every major step in the evolutionary history of plants highlights the role of water availability as a driver of plant evolution. Here the results demonstrate that gene novelty, expansion and cooption contribute differently to distinct steps in the evolution of water regulatory traits. Some of these genes evolved at the same time as the morphological innovation they are associated with (i.e. stomatal development genes), while others are older, indicating that cooption was concomitant with the evolution of these traits. Overall, our analyses shed new light on the genetic basis of the evolution of life on land, highlighting the role of genome dynamics in the diversification of the plant kingdom.

## Author contributions

AMCB, JP and UB designed the study and analyses. AMCB performed the analyses. AMCB, JP and UB wrote the manuscript.

## Supporting information


**Dataset S1** Charophyte genome Blast.

**Dataset S2** Gene occupancy.

**Dataset S3** Fasta alignments and phylogenetic trees.

**Dataset S4** List of novel, duplicated and coopted genes.

**Fig. S1** Heatmap displaying absence, partial presence and presence in all species for both transcriptomic and genomic data for genes involved in stomatal development.
**Fig. S2** Heatmap displaying absence, partial presence and presence in all species for both transcriptomic and genomic data for genes involved in stomatal signalling.
**Fig. S3** Heatmap displaying absence, partial presence and presence in all species for both transcriptomic and genomic data for genes involved in vascular tissue development.
**Fig. S4** Heatmap displaying absence, partial presence and presence in all species for both transcriptomic and genomic data for genes involved in root hair development.
**Fig. S5** Heatmap displaying absence, partial presence and presence in all species for both transcriptomic and genomic data for genes involved in primary root development.
**Fig. S6** Heatmap displaying absence, partial presence and presence in all species for both transcriptomic and genomic data for genes involved in lateral root development.
**Table S1** Additional genome data used in this study and sources of genome data.
Please note: Wiley Blackwell are not responsible for the content or functionality of any Supporting Information supplied by the authors. Any queries (other than missing material) should be directed to the *New Phytologist* Central Office.

## Data Availability

The data that support the findings of this study are available in the Supporting Information of this article.
