# Supplementary material for: Water‐related innovations in land plants evolved by different patterns of gene cooption and novelty
Source: New Phytol. 2022 Feb 8;235(2):732–42. doi: 10.1111/nph.17981 (PMC9303528; doi:10.1111/nph.17981)
Supplement: Supplementary file 1 — Dataset S1 Charophyte genome Blast. [file NPH-235-732-s003.zip › Supplementary_Data_1_charophyte_genome_blast/Supplemental Data 1 Information.docx]

Supplemental Data 1: Charophyte genome BLAST

Water-related innovations in land plants evolved by different patterns of gene co-option and novelty

Alexander M. C. Bowles, Jordi Paps, Ulrike Bechtold

Acceptance date: 25 December 2021
