# Supplementary material for: Water‐related innovations in land plants evolved by different patterns of gene cooption and novelty
Source: New Phytol. 2022 Feb 8;235(2):732–42. doi: 10.1111/nph.17981 (PMC9303528; doi:10.1111/nph.17981)
Supplement: Supplementary file 5 — Fig. S1 Heatmap displaying absence, partial presence and presence in all species for both transcriptomic and genomic data for genes involved in stomatal development. Fig. S2 Heatmap displaying absence, partial presence and presence in all species for both transcriptomic and genomic data for genes involved in stomatal signalling. Fig. S3 Heatmap displaying absence, partial presence and presence in all species for both transcriptomic and genomic data for genes involved in vascular tissue development. Fig. S4 Heatmap displaying absence, partial presence and presence in all species for both transcriptomic and genomic data for genes involved in root hair development. Fig. S5 Heatmap displaying absence, partial presence and presence in all species for both transcriptomic and genomic data for genes involved in primary root development. Fig. S6 Heatmap displaying absence, partial presence and presence in all species for both transcriptomic and genomic data for genes involved in lateral root development. Table S1 Additional genome data used in this study and sources of genome data. Please note: Wiley Blackwell are not responsible for the content or functionality of any Supporting Information supplied by the authors. Any queries (other than missing material) should be directed to the New Phytologist Central Office. [file NPH-235-732-s001.pdf]

New Phytologist Supplementary Information

Water-related innovations in land plants evolved by different patterns of gene co-option and novelty

Alexander M.C. Bowles, Jordi Paps, and Ulrike Bechtold

Acceptance date: 25 December 2021

Table S1. Additional genome data used in this study and sources of genome data.

Group	Genome	Genome paper/project
Streptophyte algae	*Chlorokybus atmophyticus*	https://doi.org/10.1038/s41477-019-0560-3
	Mesostigma viride	https://doi.org/10.1038/s41477-019-0560-3
	Chara braunii	https://doi.org/10.1016/j.cell.2018.06.033
	Penium margaritaceum	https://doi.org/10.1016/j.cell.2020.04.019
	Spirogloea muscicola	https://doi.org/10.1016/j.cell.2019.10.019
	Mesotaenium endlicherianum	https://doi.org/10.1016/j.cell.2019.10.019
Liverworts	*Anthoceros angustus*	https://doi.org/10.1038/s41477-019-0588-4
	Anthoceros agrestis	https://doi.org/10.1038/s41477-020-0618-2
	Anthoceros punctactus	https://doi.org/10.1038/s41477-020-0618-2
Mosses	*Fontinalis antipyretica*	https://doi.org/10.46471/gigabyte.8
	Pleurozium schreberi	https://doi.org/10.1534/g3.119.400279
Ferns	*Azolla filiculoides*	https://doi.org/10.1038/s41477-018-0188-8
	Salvinia cucullata	https://doi.org/10.1038/s41477-018-0188-8
Gymnosperms	*Abies alba*	https://doi.org/10.1534/g3.119.400083
	Picea glauca	https://doi.org/10.1093/bioinformatics/btt178
	Picea lambertiana	https://doi.org/10.1534/genetics.116.193227
	Picea taeda	https://doi.org/10.1186/gb-2014-15-3-r59
	Pseudotsuga menziesii	https://doi.org/10.1534/g3.117.300078
	Sequoiadendron giganteum	https://doi.org/10.1534/g3.120.401612

Birol I, Raymond A, Jackman SD, Pleasance S, Coope R, Taylor GA, Yuen MM Saint, Keeling CI, Brand D, Vandervalk BP, *et al.* 2013. Assembling the 20 Gb white spruce (Picea glauca) genome from whole-genome shotgun sequencing data. *Bioinformatics (Oxford, England)* **29**: 1492–7.

Cheng S, Xian W, Fu Y, Marin B, Keller J, Wu T, Sun W, Li X, Xu Y, Zhang Y, *et al.* 2019. Genomes of Subaerial Zygnematophyceae Provide Insights into Land Plant Evolution. *Cell* **179**: 1057–1067.

Jiao C, Sørensen I, Sun X, Sun H, Behar H, Alseekh S, Philippe G, Palacio Lopez K, Sun L, Reed R, *et al.* 2020. The Penium margaritaceum Genome: Hallmarks of the Origins of Land Plants. *Cell* **181**: 1097–1111.

Li F-W, Brouwer P, Carretero-Paulet L, Cheng S, de Vries J, Delaux P-M, Eily A, Koppers N, Kuo L-Y, Li Z, *et al.* 2018. Fern genomes elucidate land plant evolution and cyanobacterial symbioses. *Nature Plants* **4**: 460–472.

Li F, Nishiyama T, Waller M, Frangedakis E, Keller J, Li Z, Fernandez-Pozo N, Barker MS, Bennett T, Blázquez MA, *et al.* 2020. Anthoceros genomes illuminate the origin of land plants and the unique biology of hornworts. *Nature Plants* **6**: 259–272.

Mosca E, Cruz F, Gómez-Garrido J, Bianco L, Rellstab C, Brodbeck S, Csilléry K, Fady B, Fladung M, Fussi B, *et al.* 2019. A reference genome sequence for the european silver fir (Abies alba Mill.): A community-generated genomic resource. *G3: Genes, Genomes, Genetics* **9**: 2039–2049.

Neale DB, McGuire PE, Wheeler NC, Stevens KA, Crepeau MW, Cardeno C, Zimin A V., Puiu D, Pertea GM, Sezen UU, *et al.* 2017. The Douglas-Fir genome sequence reveals specialization of the photosynthetic apparatus in Pinaceae. *G3: Genes, Genomes, Genetics* **7**: 3157–3167.

Nishiyama T, Sakayama H, de Vries J, Buschmann H, Saint-Marcoux D, Ullrich KK, Haas FB, Vanderstraeten L, Becker D, Lang D, *et al.* 2018. The Chara Genome: Secondary Complexity and Implications for Plant Terrestrialization. *Cell* **174**: 448–464.

Pederson ERA, Warshan D, Rasmussen U. 2019. Genome sequencing of Pleurozium schreberi: The assembled and annotated draft genome of a pleurocarpous feather moss. *G3: Genes, Genomes, Genetics* **9**: 2791–2797.

Scott AD, Zimin A V., Puiu D, Workman R, Britton M, Zaman S, Caballero M, Read AC, Bogdanove AJ, Burns E, *et al.* 2020. A Reference Genome Sequence for Giant Sequoia. *G3: Genes, Genomes, Genetics* **10**: 3907–3919.

Stevens KA, Wegrzyn JL, Zimin A, Puiu D, Crepeau M, Cardeno C, Paul R, Gonzalez-Ibeas D, Koriabine M, Holtz-Morris AE, *et al.* 2016. Sequence of the Sugar Pine Megagenome. *Genetics* **204**: 1613–1626.

Wang S, Li L, Li H, Sahu SK, Wang H, Xu Y, Xian W, Song B, Liang H, Cheng S, *et al.* 2019. Genomes of early-diverging streptophyte algae shed light on plant terrestrialization. *Nature Plants* **6**: 95–106.

Yu J, Li L, Wang S, Dong S, Chen Z, Patel N, Goffinet B, Chen H, Liu H, Liu Y. 2020. Draft genome of the aquatic moss Fontinalis antipyretica (Fontinalaceae, Bryophyta). *Gigabyte* **2020**: 1–9.

Zhang J, Fu XX, Li RQ, Zhao X, Liu Y, Li MH, Zwaenepoel A, Ma H, Goffinet B, Guan YL, *et al.* 2020. The hornwort genome and early land plant evolution. *Nature Plants* **6**: 107–118.

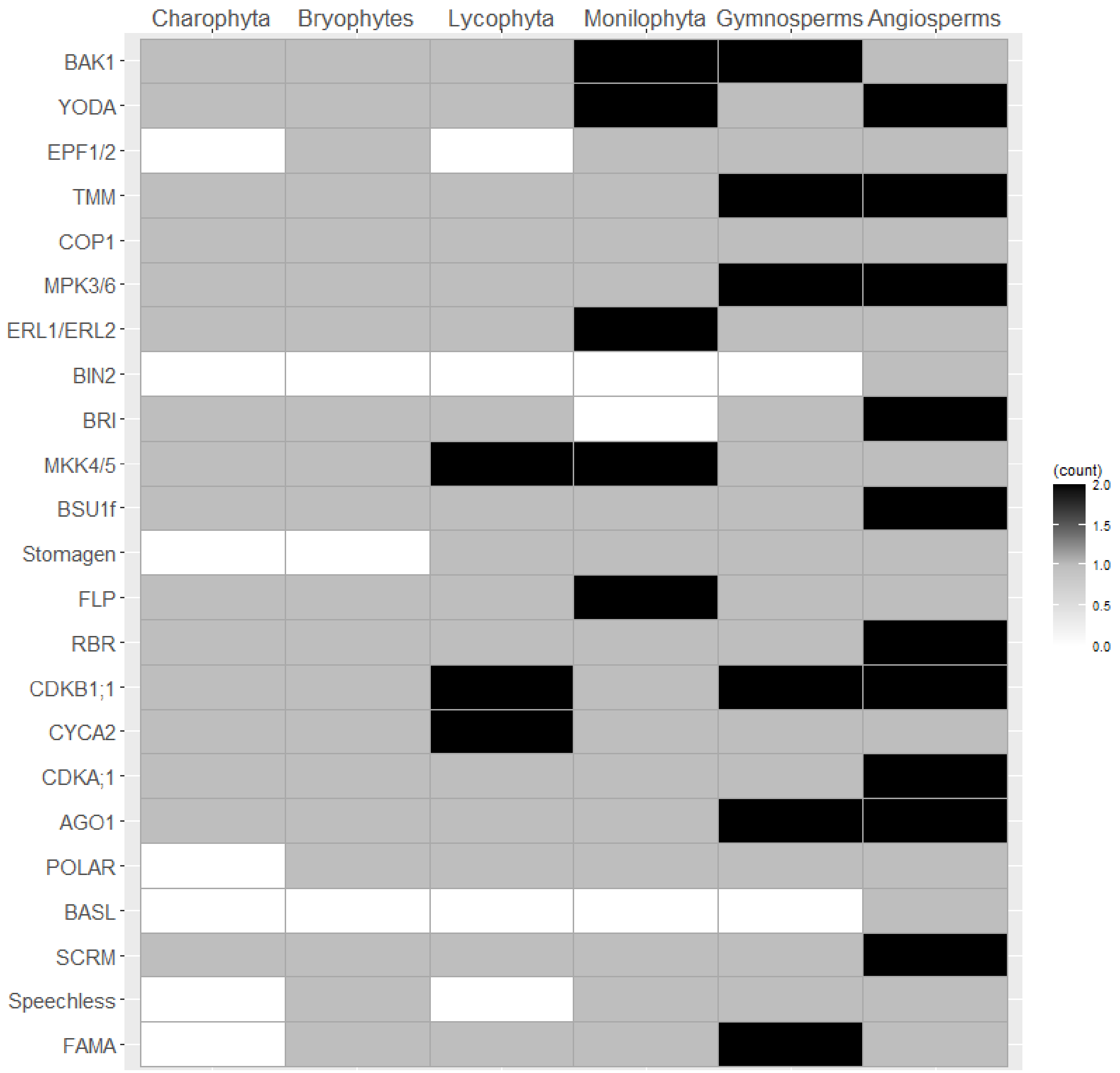

Fig. S1. Heatmap displaying absence (white), partial presence (grey) and presence (black) in all species for both transcriptomic and genomic data for genes involved in stomatal development.

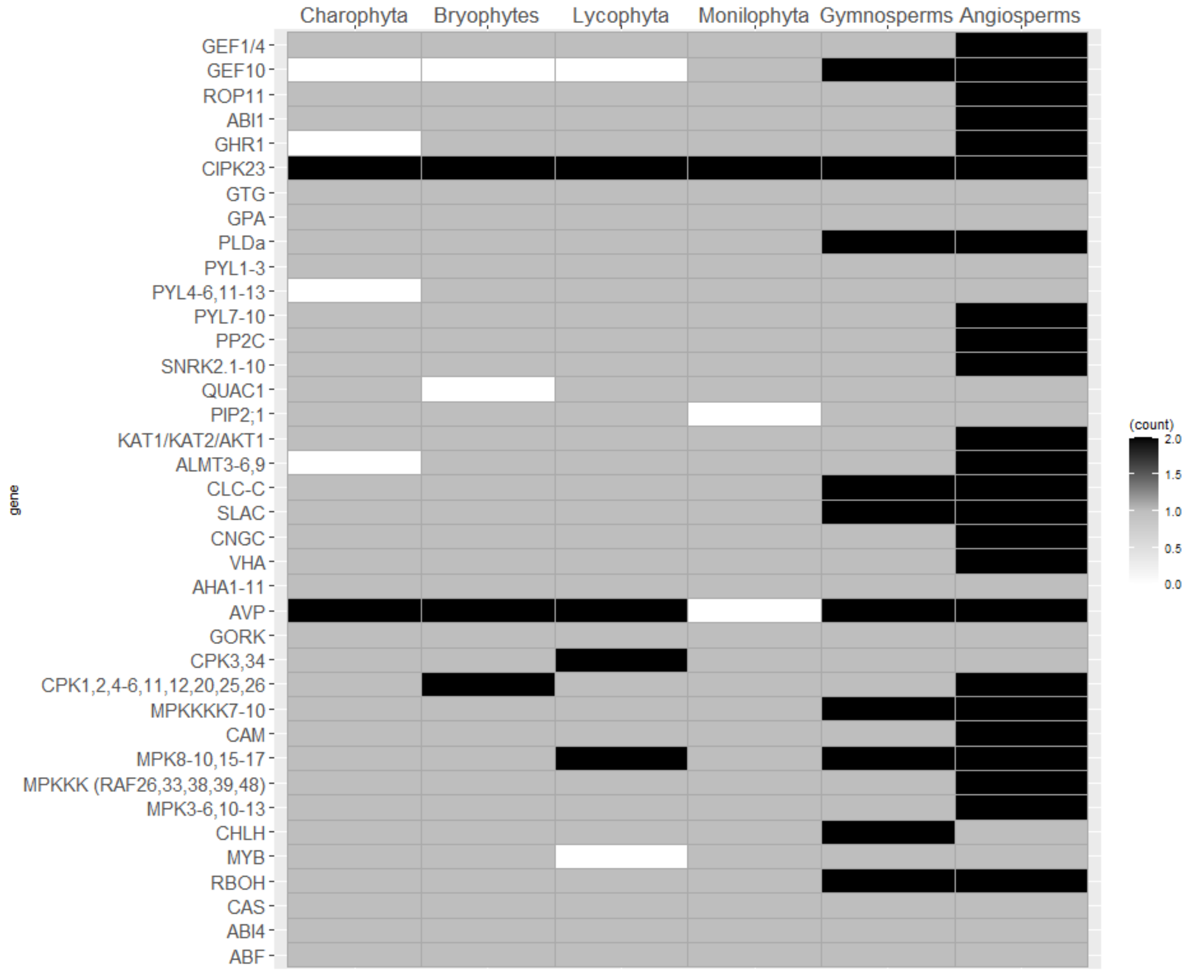

Fig. S2. Heatmap displaying absence (white), partial presence (grey) and presence (black) in all species for both transcriptomic and genomic data for genes involved in stomatal signalling.

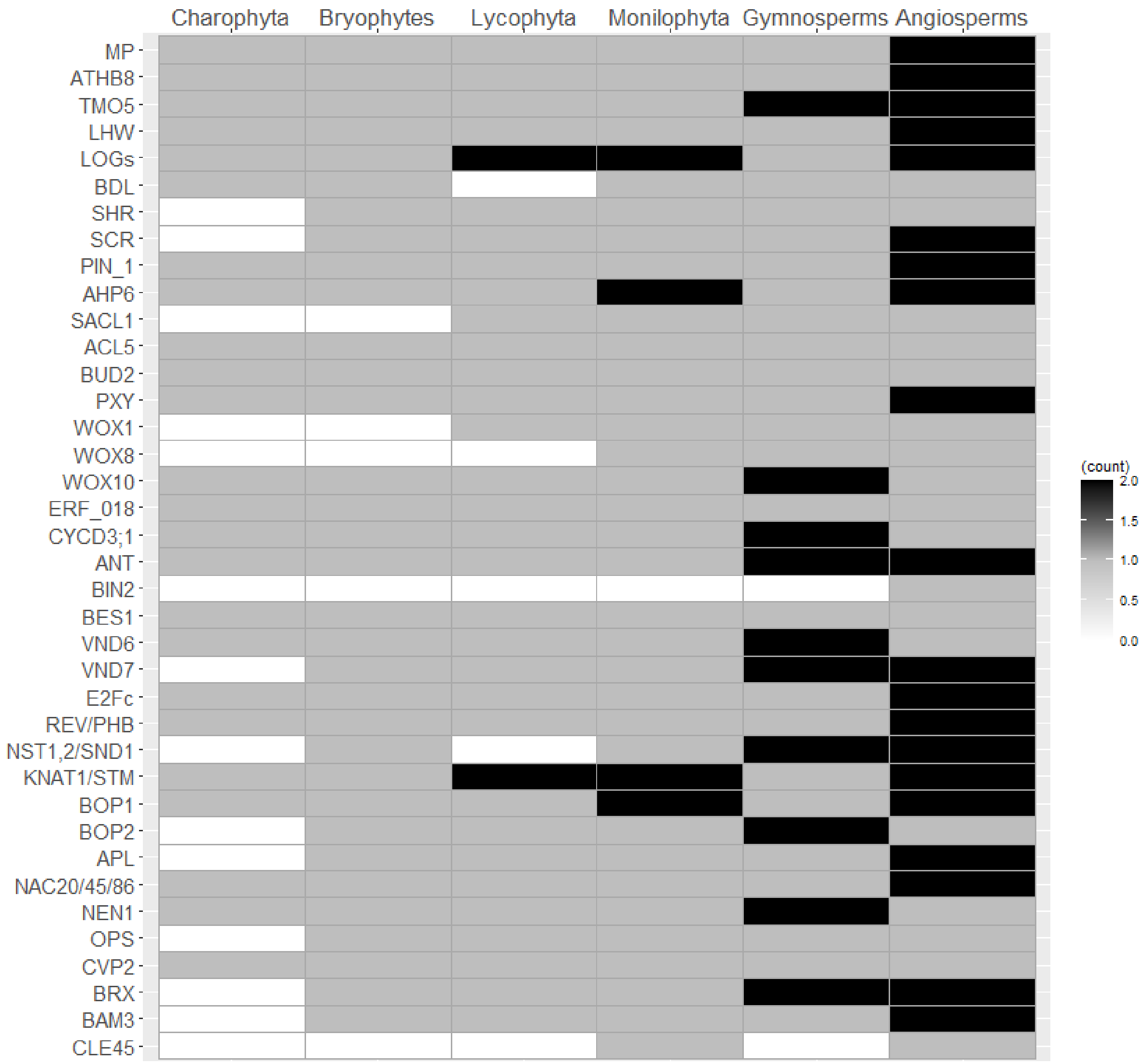

Fig. S3. Heatmap displaying absence (white), partial presence (grey) and presence (black) in all species for both transcriptomic and genomic data for genes involved in vascular tissue development.

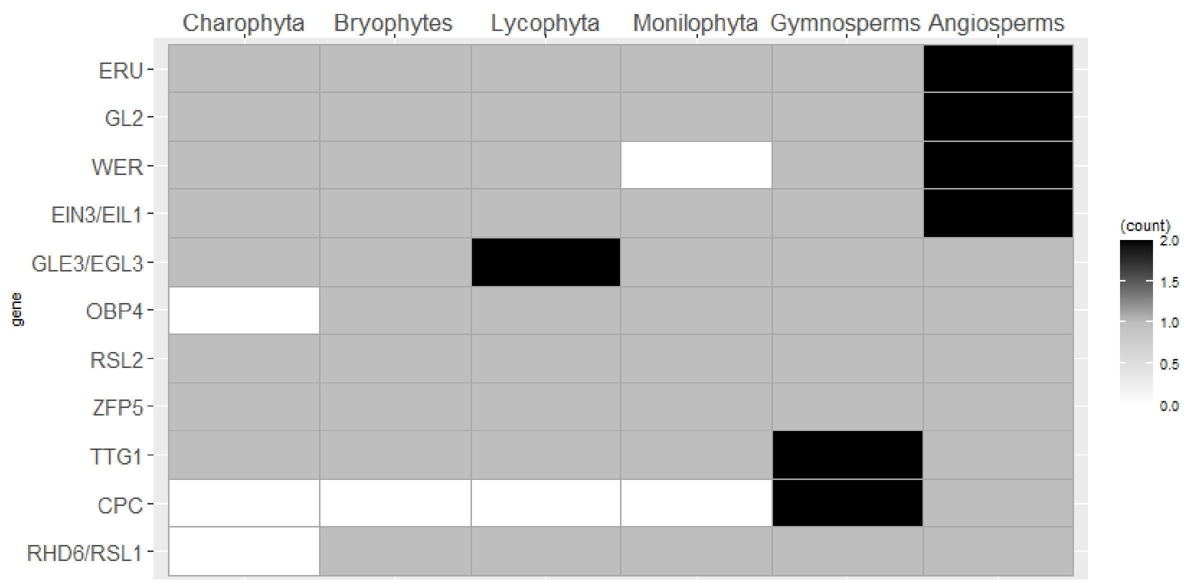

Fig. S4. Heatmap displaying absence (white), partial presence (grey) and presence (black) in all species for both transcriptomic and genomic data for genes involved in root hair development.

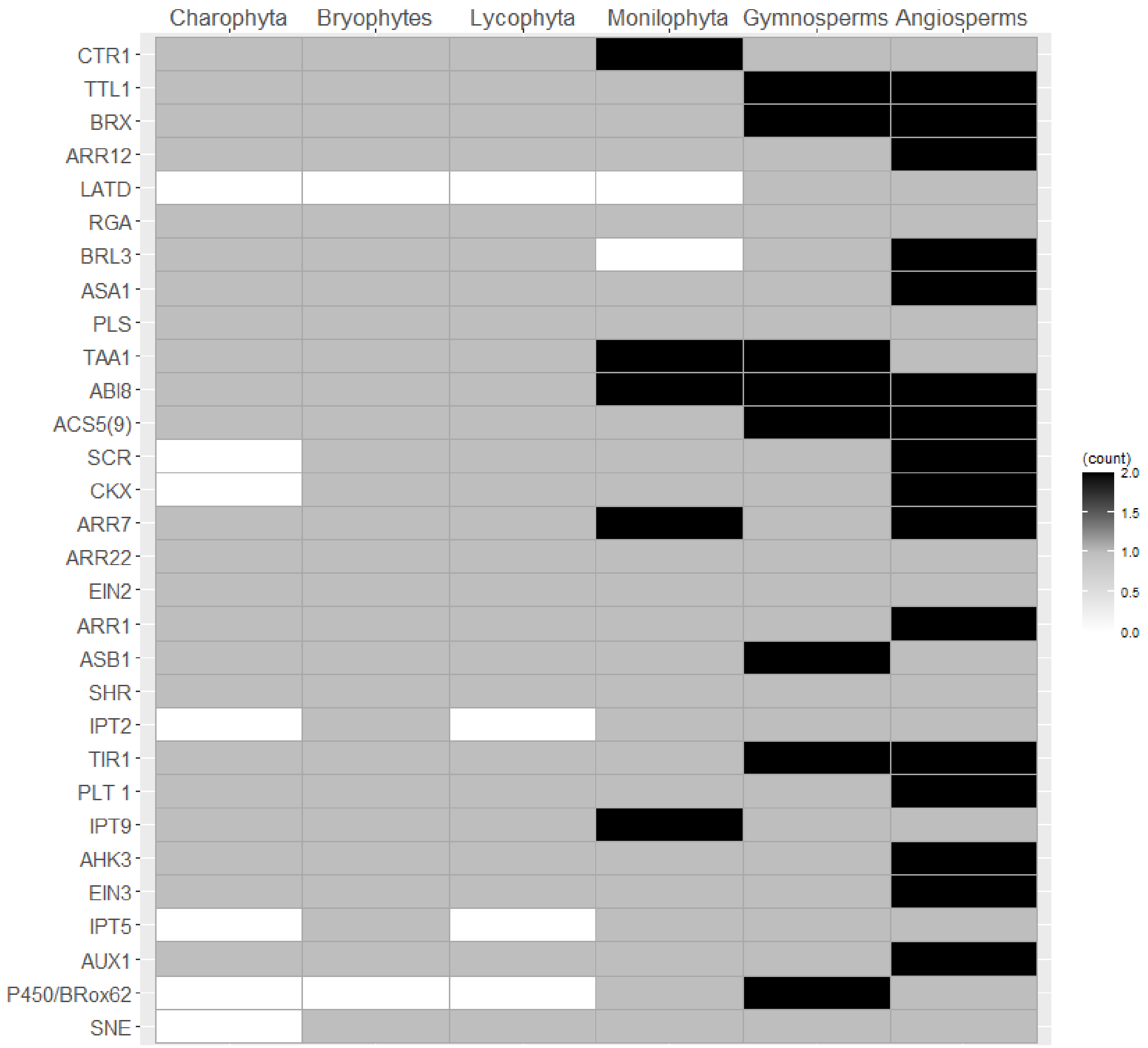

Fig. S5. Heatmap displaying absence (white), partial presence (grey) and presence (black) in all species for both transcriptomic and genomic data for genes involved in primary root development.

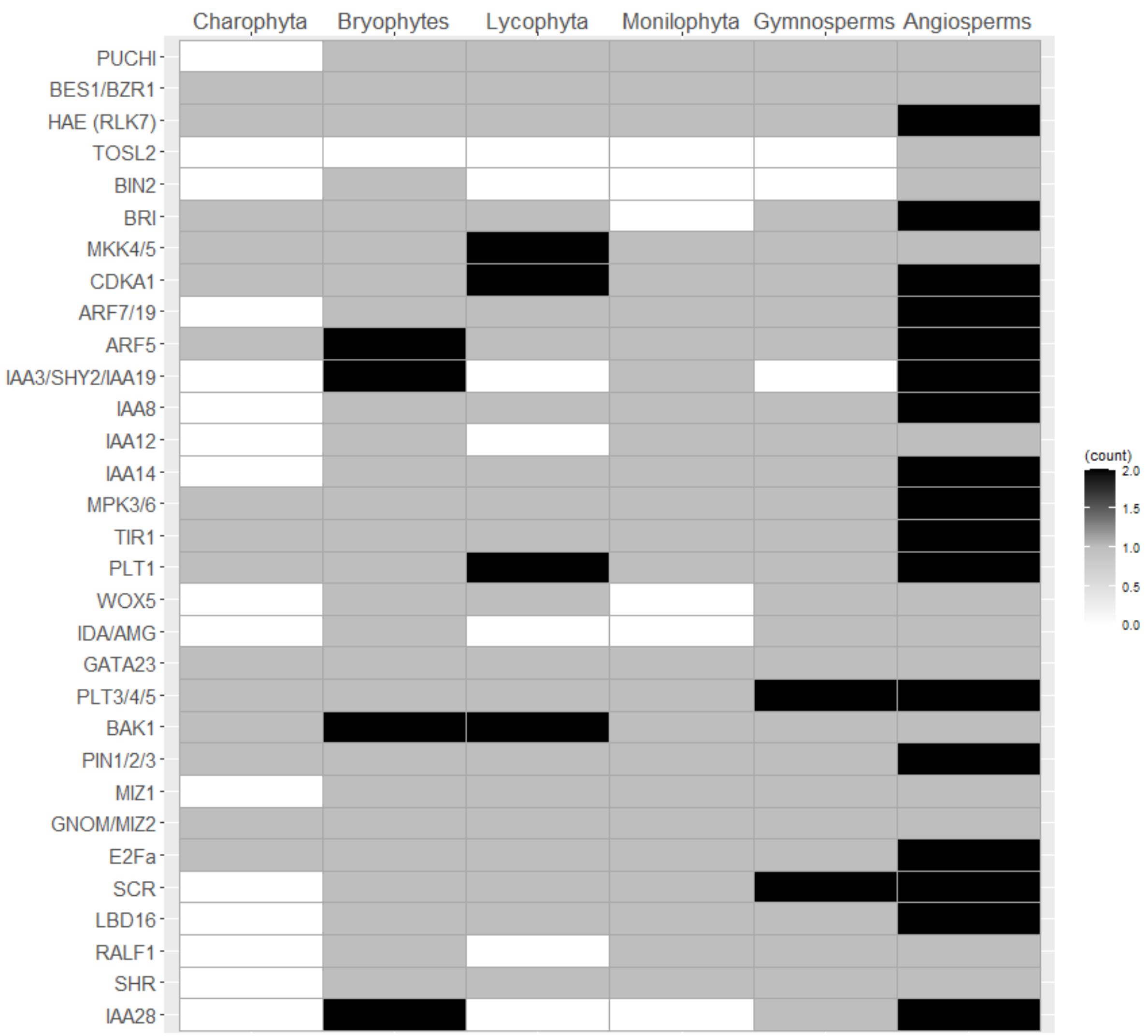

Fig. S6. Heatmap displaying absence (white), partial presence (grey) and presence (black) in all species for both transcriptomic and genomic data for genes involved in lateral root development.

Supplementary Datasets

Supplemental Data 1: Charophyte genome BLAST (see separate file)

Supplemental Data 2: Gene occupancy (see separate file)

Supplemental Data 3: FASTA alignments and phylogenetic trees (see separate file)

Supplemental Data 4: List of novel, duplicated and co-opted genes (see separate file)
