# Supplementary material for: Water‐related innovations in land plants evolved by different patterns of gene cooption and novelty
Source: New Phytol. 2022 Feb 8;235(2):732–42. doi: 10.1111/nph.17981 (PMC9303528; doi:10.1111/nph.17981)
Supplement: Supplementary file 3 — Dataset S3 Fasta alignments and phylogenetic trees. [file NPH-235-732-s005.zip › Supplementary_Data_3_fasta_alignments_trees/Supplemental Data 3 Information.docx]

Supplemental Data 3: FASTA alignments and phylogenetic trees

Water-related innovations in land plants evolved by different patterns of gene co-option and novelty

Alexander M. C. Bowles, Jordi Paps, Ulrike Bechtold

Acceptance date: 25 December 2021
